# Tertiary lymphoid structural heterogeneity determines tumour immunity and prospects for clinical application

**DOI:** 10.1186/s12943-024-01980-6

**Published:** 2024-04-06

**Authors:** Yuyuan Zhang, Mengjun Xu, Yuqing Ren, Yuhao Ba, Shutong Liu, Anning Zuo, Hui Xu, Siyuan Weng, Xinwei Han, Zaoqu Liu

**Affiliations:** 1 Department of Interventional Radiology, The First Affiliated Hospital of Zhengzhou University, Zhengzhou, Henan 450052 China; 2 Medical School of Zhengzhou University, Zhengzhou, Henan China; 3 Department of Respiratory and Critical Care Medicine, The First Affiliated Hospital of Zhengzhou University, Zhengzhou, Henan 450052 China; 4 Interventional Institute of Zhengzhou University, Zhengzhou, Henan 450052 China; 5 Interventional Treatment and Clinical Research Center of Henan Province, Zhengzhou, Henan 450052 China; 6 Institute of Basic Medical Sciences, Chinese Academy of Medical Sciences and Peking Union Medical College, Beijing, 100730 China

**Keywords:** Tertiary lymphoid structures, Tumour immunity, Cellular crosstalk, Biomarkers, Biomaterials

## Abstract

Tertiary lymphoid structures (TLS) are clusters of immune cells that resemble and function similarly to secondary lymphoid organs (SLOs). While TLS is generally associated with an anti-tumour immune response in most cancer types, it has also been observed to act as a pro-tumour immune response. The heterogeneity of TLS function is largely determined by the composition of tumour-infiltrating lymphocytes (TILs) and the balance of cell subsets within the tumour-associated TLS (TA-TLS). TA-TLS of varying maturity, density, and location may have opposing effects on tumour immunity. Higher maturity and/or higher density TLS are often associated with favorable clinical outcomes and immunotherapeutic response, mainly due to crosstalk between different proportions of immune cell subpopulations in TA-TLS. Therefore, TLS can be used as a marker to predict the efficacy of immunotherapy in immune checkpoint blockade (ICB). Developing efficient imaging and induction methods to study TA-TLS is crucial for enhancing anti-tumour immunity. The integration of imaging techniques with biological materials, including nanoprobes and hydrogels, alongside artificial intelligence (AI), enables non-invasive in vivo visualization of TLS. In this review, we explore the dynamic interactions among T and B cell subpopulations of varying phenotypes that contribute to the structural and functional diversity of TLS, examining both existing and emerging techniques for TLS imaging and induction, focusing on cancer immunotherapies and biomaterials. We also highlight novel therapeutic approaches of TLS that are being explored with the aim of increasing ICB treatment efficacy and predicting prognosis.

## Introduction

Effective anti-tumour immunity necessitates the presence of tumour-infiltrating lymphocytes (TILs). This has been particularly evidenced by the identification of tertiary lymphoid structures (TLS), representing well-organized clusters of TILs that elicit delayed immune responses [1]. TLS, immune cell clusters akin to secondary lymphoid organs (SLOs), emerge postnatally within non-lymphoid tissues, typically absent under normal physiological conditions, and predominantly manifest in chronic inflammatory conditions, including cancer, chronic infections, and autoimmune diseases [1, 2]. The context of chronic inflammation is strongly associated with tumour development. Analyses of lymphocyte infiltration and the spatial aggregation of TLS within the tumour microenvironment (TME) of patients with colorectal cancer (CRC), non-small-cell lung cancer (NSCLC), breast cancer (BC), and melanoma indicate that a patient’s prognosis correlates with a higher degree of lymphocyte infiltration, density, and maturation [3–6]. Evaluating the maturity of TLS across different tumour types is critical for establishing TLS as a reliable prognostic tool in oncology. In addition, inducing a higher density and maturity of TLS in cancer patients contributes to the enhancement of the body’s anti-tumour immunity. Furthermore, promoting a greater density and maturity of TLS in cancer patients significantly enhances the body’s anti-tumour immune response.

In recent decades, immunotherapy targeting immune checkpoints—specifically programmed cell death protein 1 (PD-1), and its ligands programmed death ligand 1 (PD-L1) and cytotoxic T-lymphocyte-associated protein 4 (CTLA-4)—has emerged as a pivotal strategy against various solid tumours. Nevertheless, immune checkpoint blockade (ICB) faces challenges, including clinical application limitations and safety concerns, potentially resulting in inflammatory side effects or immune-related adverse events (irAEs) [7, 8]. Intratumoral TLS has gained recognition as a biomarker capable of predicting and potentially enhancing ICB therapy efficacy [9, 10]. Analyses of intratumoral TLS have demonstrated a positive correlation with clinical benefits in patients, irrespective of PD-L1 expression status, facilitating new markers’ use in ICB therapy patient selection [11]. The distinct features of nanoparticles in multimodal and molecular imaging enable them to be utilized for extremely delicate non-invasive multimodal imaging of biological occurrences in vivo [12]. Developing more efficient, non-invasive techniques for visualizing and inducing TLS represents a novel research direction [13].

In this review, we outline the tissue and cellular structure of TLS and explore the pathways and molecular mechanisms involved in TLS formation and maturation. The dual effects of immune cell crosstalk within TLS on anti-tumour immunity are described, and the potential of TLS as a cancer biomarker is highlighted. Finally, the current induction strategies for TLS are summarized, and the dilemmas and solutions for its future development are discussed.

## Development and characterization of mature TLS

Based on the impact of mature TLS on tumour prognosis and treatment, it is important to understand the mechanisms of immune cell crosstalk within the internal tissue and cellular structure of mature TLS in the subsequent anti-tumour immune response. It is also essential to understand the important mechanisms of action during TLS formation and maturation.

### Composition of mature TLS

The mature tertiary lymphoid structure is an internal region found in lymphoid follicles formed by CD20 + B cells that are surrounded by CD3 + T cells. This structure is similar to a secondary lymphoid organ (SLO). It comprises mainly of T cells, B cells, follicular dendritic cells (FDCs), dendritic cells (DCs), stromal cells, macrophages, endothelial cells, and high endothelial venules (HEVs). Presently, the T-cell region within the TLS has been thoroughly researched, being recognized as a biomarker for clinical outcome prediction [14, 15]. Characterization of TLS composition in 39 intrahepatic cholangiocarcinoma (iCCA) samples using multiplex immunohistochemistry (mIHC) revealed that the spatial distribution and abundance of TLS were significantly correlated with prognosis, and T follicular helper (Tfh) cells and regulatory T (Treg) cells may play a key role in determining the functional localization of spatially distinct TLS [16]. The function of B cells in tumour immunity has emerged as a focal point of recent research [9, 17, 18]. By employing single-cell technologies, immunohistochemistry (IHC) and immunofluorescence (IF) analyses, distinctive subpopulations of B cells have been discerned in human tumours, identified by phenotypic markers for which a general consensus has been reached, and numerous phenotypes have been found to play disparate roles in tumour prognosis [19]. However, it is important to note that the function of B cells within TLS is affected by the degree of TLS maturation. In order to achieve a complete anti-tumour immune response, immunoglobulin class switching and affinity maturation are necessary. Ayana T Ruffin and colleagues demonstrated that in human papillomavirus (HPV)-induced head and neck squamous cell carcinoma (HNSCC), tumour-infiltrating B-cell (TIL-B) transcriptional profiles, as well as spatial organization of TLS immune cells compatible with germinal centers (GCs), bear a significant association with a favorable prognosis in patients [20]. The induction of TLS in a mouse model of HPV HNSCC was found to bolster the response to PD-1 blockade therapy, which was weakened by CD20 + B cell elimination. These studies have shown that improving TIL-B cell responses could serve as a complementary approach to T cell-mediated immunotherapy. There is potential for new ideas and research directions regarding the roles of T and B cells in TLS in combating tumours. The focus is on exploring potential mechanisms of immune cell crosstalk in mature TLS.

### Formation and maturation of TLS

**Fig. 1:**
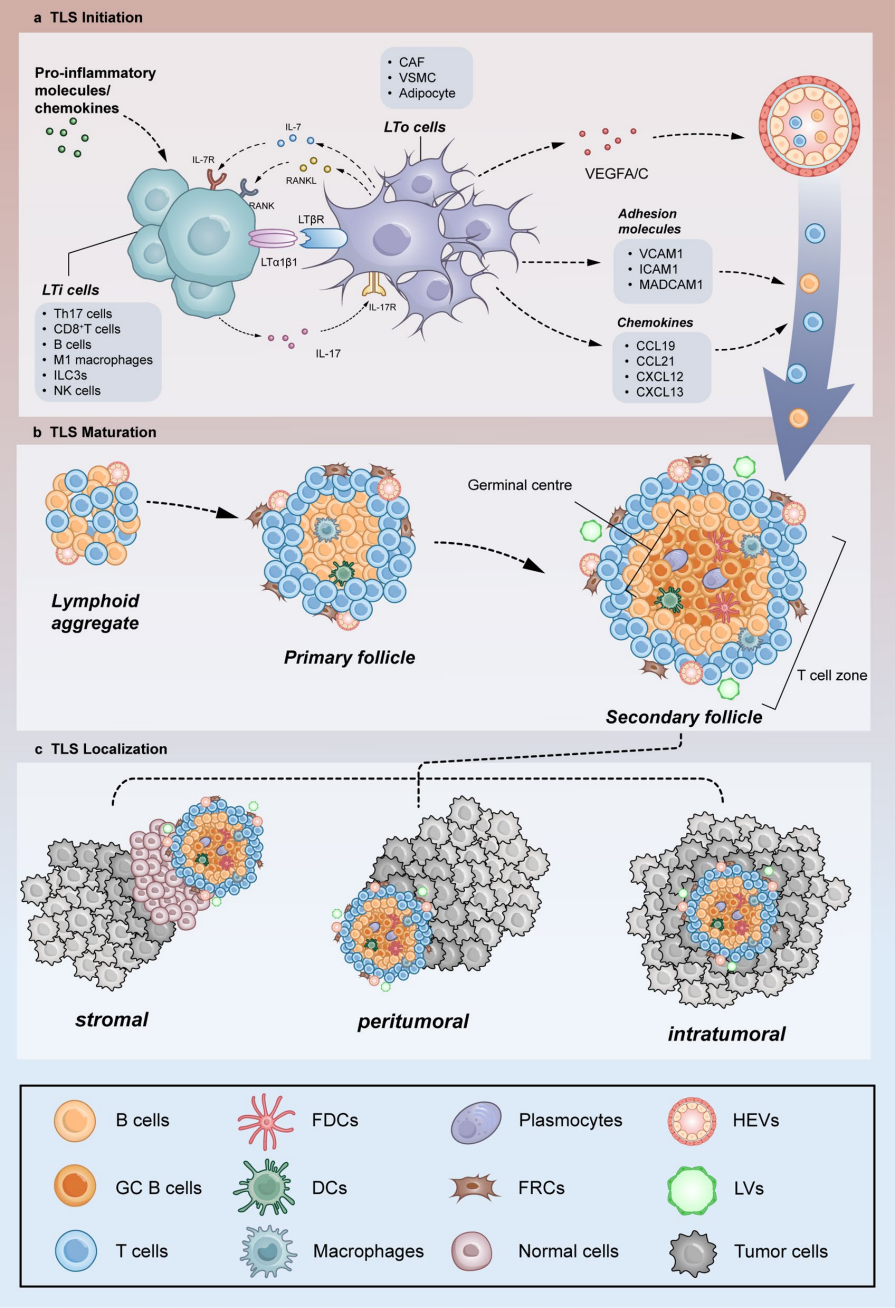
TLS formation modelling. (**a**) The initial process of TLS. Local accumulation of pro-inflammatory molecules and chemokines can recruit lymphoid tissue-inducing (LTi) cells to sites of inflammation. LTi cells interact with local lymphoid tissue organizer (LTo) cells to initiate expression of various cytokines (IL-7, IL-17 and RANKL) and cytokine receptors (IL-7R, IL-17R and RANK) and TLS development. Many immune cells can replace LTi cells, such as T helper 17 (Th17) cells, CD8 + T cells, B cells, M1-polarized macrophages, innate lymphoid cell-3(ILC3) and natural killer cells. Similarly, cancer-associated fibroblast (CAF), vascular smooth muscle cells (VAMC) and adipocyte can replace LTo cells. Through the binding of lymphotoxin α1β2 (LTα1β2) to LTβ receptor (LTβR), this signaling pathway promotes the secretion of vascular endothelial growth factor A (VEGFA) and vascular endothelial growth factor C (VEGFC) to promote HEV development. It can also promote the secretion of adhesion molecules used to recruit immune cells, such as vascular adhesion molecule 1 (VCAM 1), intercellular adhesion molecule 1 (ICAM1), and mucosal vascular addressin cell adhesion molecule 1 (MADCAM 1) as well as chemokines (CCL19, CCL21, CXCL12, and CXCL13). These chemokines induce LTα1β2 expression on lymphocytes, recruit lymphocytes from nearby HEVs into TLS and form T-cell and B-cell zones. (**b**) The maturity stage of TLS. Maturation of TLS undergoes three stages of evolution. From loose lymphoid aggregates to primary follicles of T and B cells with macrophages and DCs, to mature polarized structures containing GCs, host cells, HEVs, and lymph vessels (LVs). (**c**) The location of TLS in the body. The location of TLS in the human body includes stromal, peritumoral and intratumoral. IL-7, interleukin-7; RANK, NF-κB receptor activator; RANKL, NF-κB ligand receptor activator; CCL19, C-C motif chemokine 19; CXCL12, C-X-C motif chemokine 12

TLS and SLO share similarities, making it possible to study TLS by exploring the developmental processes of SLO. Nevertheless, the cellular components and molecular pathways involved in the initiation and developmental processes of TLS vary due to different tissue origins and disease states, and their intrinsic functional properties differ from those of SLO (Fig. 1a; Table 1) [21]. The formation of TLS depends on intricate ligand-receptor interactions between different cell types, including innate lymphocytes (ILCs), stromal cells, endothelial cells and B cells. CD3-CD4-/+ CXCR5IL-7Rα + hi ILCs, also known as lymphoid tissue inducing cells (LTi), are an essential component of SLO development [22]. Stromal fibroblasts, characterized by the expression of podoplanin and fibroblast activation protein-α (FAP), have been shown to be associated with the formation of TLS in both mouse and human models [23, 24]. The secretion of IL-7 and CXCL13 by stromal fibroblasts recruits and induces LTi cell proliferation and expansion, contributing to the development of SLOs. Subsequent secretion of lymphotoxin α1β2 (LTα1β2) by LTi cells induces maturation of lymphoid tissue-forming cells (LTo), leading to up-regulation of CXCL13, CCL19 and CCL21, which promotes the infiltration of B and T cells into the TLS [9, 25, 26]. Additionally, HEVs are involved in the process of TLS maturation by promoting intratumoral lymphocyte aggregation and infiltration, creating an environment conducive to further TLS maturation [27]. Following TLS maturation, activated immune cells transit from TLS to the tumour bed through HEVs, thereby exerting potent anti-tumour immunity against cancer cells [28].

**Table 1:** Comparison between secondary lymphoid organs and tertiary lymphoid structures

Characteristics	Secondary lymphoid organs	Tertiary lymphoid structures	References
Examples	Spleen, regional lymph nodes, peyer’s patches, ILFs, tonsils and NALT	Chronic Infection, autoimmune diseases, transplant rejection, cancer	[29–34]
Origin	During embryogenesis	After birth	[29, 35, 36]
Induction and development	LTo (FAP + podoplanin+, LTβR+), LTi, endothelial cells, retinoic acid	LTo (FAP + podoplanin+, LTβR+), ILC3/LTi, endothelial cells, NKT, Th17, chronic inflammation, cytokines (LT, TNF, LIGHT, IL-17), chemokines (CXCL13, CCL19, CCL21)	[10, 37–40]
Location	Defined-relatively immutable	Variable (non-lymphoid organ or tissue site)	[29, 35, 41, 42]
Structure	B and T cell compartments, HEVs, lymphatic vessels	Variable (from mixed T and B cells to T and B cell compartments), HEVs, lymphatic vessels	[2, 35, 36, 42, 43]
Capsule	Defined	Rare	[2, 36, 42]
Self-tolerance	Maintained	Disturbed (autoantibody production etc.)	[2, 10, 44, 45]
Durability	Permanent (but can collapse)	Transient	[39]

ILFs: isolated lymphoid follicle; NALT: nasal associated lymphoid tissue; FAP: fibroblast activation protein-α; ILC3: innate lymphocyte 3; NKT: natural killer T cells; LTs: leukotrienes; TNF: tumour necrosis factor

Mature TLSs are typically associated with positive anti-tumour immune responses in cancer. However, the role of TLS in the cancer immune landscape varies, with not all TLS actively contributing to the immune response against cancer. This variability may be attributed to differences in TLS density and maturity, which influence their structural composition and cellular makeup. The maturation of TLS is a multi-stage process, characterized by distinct structural and functional phases (Fig. 1b). Immature TLS, also known as early TLS (E-TLS), represent the initial stage, consisting of loosely aggregated T cells, B cells, and stromal cells. These early structures act as a foundation, recruiting additional immune cells and evolving into more organized forms in response to chemokines and cytokines (e.g. CXCL13, CXCL12, TNF-α, IL-17) secreted by stromal or lymphocyte cells [25, 46]. The resulting primary follicular-like TLS (PFL-TLS) feature T-cell-surrounded B-cell clusters with follicular DCs but lack GCs [25]. The transition to secondary follicle-like TLS (SFL-TLS) marks the final maturation stage, distinguished primarily by the emergence of GC activity. Mature TLS also exhibit a different positional relationship to the tumour (Fig. 1c). Despite these classifications, the efficacy of E-TLS in inducing anti-tumour responses remains unproven, and the presence of TA-TLS does not guarantee a beneficial immune response. Current evidence suggests that only TLS with GCs are functional in combating tumours [47]. Research further elucidates this complexity. Pathological and gene expression profiling of 127 early liver lesions revealed that 24% of the samples exhibited E-TLS and that these were clearly associated with immunosuppression and elevated expression of immunodeficient genes, which could not prevent progression to hepatocellular carcinoma (HCC) and appeared to favor immune evasion [48]. In another study involving 273 HCC patients, findings indicated a correlation between the risk of early tumour relapse and the presence, as well as the level of maturity, of TLS within the tumours [49]. Notably, the presence of mature TLS within tumours significantly improved the overall survival rates of patients with perihepatic cholangiocarcinoma. This outcome starkly contrasts with the effects observed in the presence of E-TLS or PFL-TLS, underscoring the critical role of TLS maturity in patient prognosis [50].

## Intercellular interaction in TLS promotes anti-tumour immunity

**Fig. 2:**
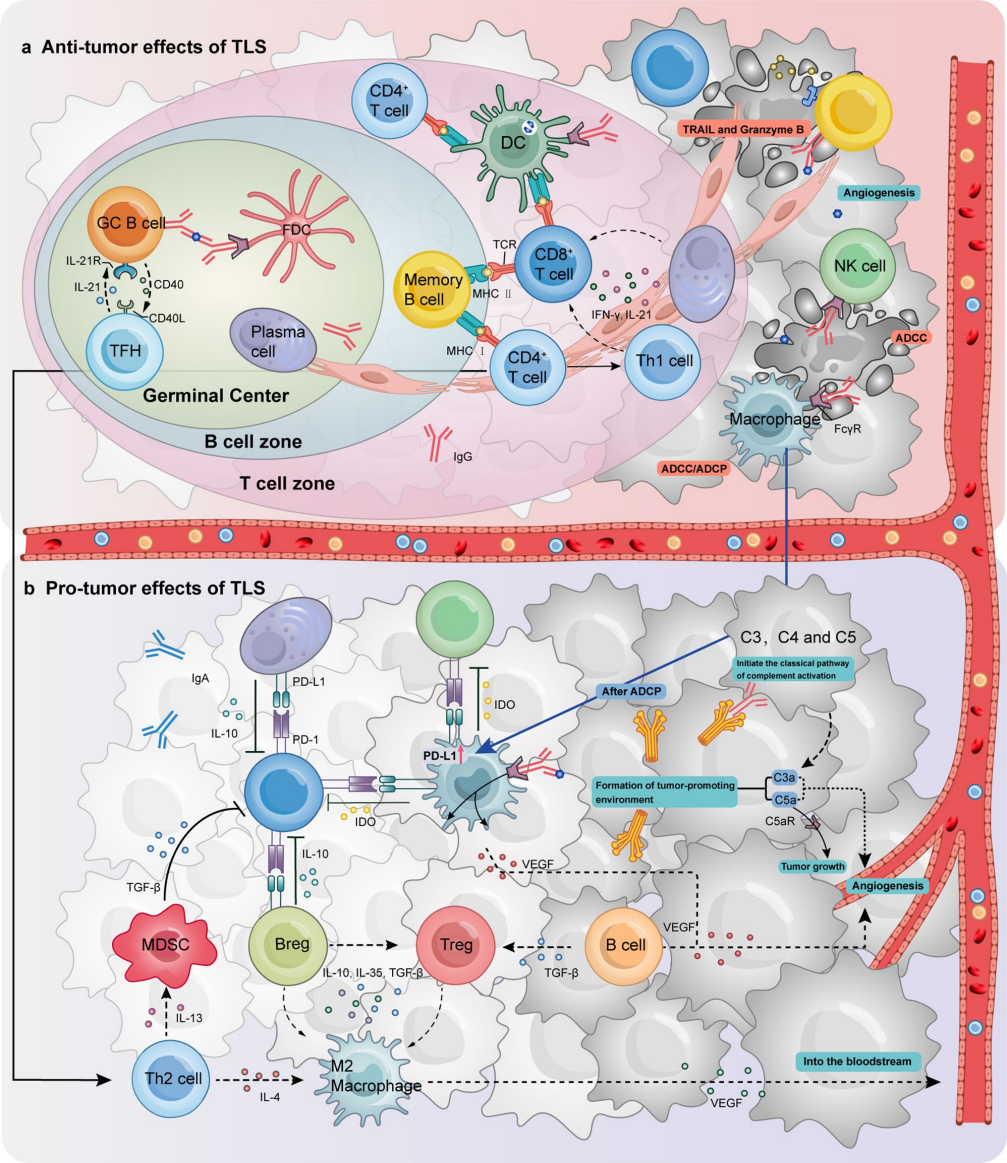
The roles of TLS in tumour immunity. (**a**) Anti-tumour effects of immune cell crosstalk in TLS. In the germinal centre (GC) of TLS, GC B cells differentiate into memory B cells and plasma cells (PCs) after receiving antigen delivery from FDCs and interacting with T follicular helper cells (Tfh). In the T-cell compartment, differentiated B cells and dendritic cells (DCs) present antigenic peptides to effector T cells, promoting infiltration of effector T cells into the tumour bed. Plasma cells follow the trajectory of fibroblasts into the tumour bed and produce anti-tumour IgG antibodies. In tumours, memory B cells can destroy tumour cells by expressing tumour necrosis factor-associated apoptosis-inducing ligand (TRAIL) or releasing granzyme B. Fc receptors on natural killer (NK) cells and macrophages can bind to a constant region of anti-tumour antibodies and destroy tumour cells through antibody-dependent cell-mediated cytotoxicity (ADCC) or antibody-dependent cell-mediated phagocytosis (ADCP). (**b**) Tumour-promoting effect of immune cell crosstalk in TLS. IgA PCs are associated with the secretion of suppressor cytokines, which generate an immunosuppressive environment favouring the emergence of M2 macrophages, myeloid-derived suppressor cells (MDSC), regulatory T (Treg) cells, and B (Breg) cells, and suppressing the function of effector T cells. Th2 cells produce IL-4 and IL-13, respectively, which promote tumour entry into the circulation by increasing epidermal growth factor expression and indirectly inhibit effector T cell function by increasing TGF-β production by MDSC. Macrophages upregulate PD-L1 to support local immunosuppression after ADCP. Complement component 1q (C1q) produced by macrophages binds to IgG-covered tumour cells, activating the classical complement cascade reaction and promoting tumour growth and angiogenesis. B Cells also produce VEGF, which promotes angiogenesis

Previous studies have highlighted that elevated densities of TILs are associated with favorable clinical outcomes across a wide array of solid tumours, underscoring the therapeutic promise of TIL-based approaches [51, 52]. Within the milieu of TIL-positive tumours, mature TLS emerge as pivotal arenas for intricate immune cell interactions [53]. These interactions within an active TLS microenvironment not only facilitate the (re)activation of initial or memory immune cells but also promote the expansion of effector cells, a process that surpasses the capabilities of TILs navigating the tumour bed without directional cues. Despite these beneficial dynamics, the accumulation of regulatory cells within TLS may dampen immune responses, leading to the potential inactivation or dormancy of TLS [54]. Nonetheless, when immune cells within TLS work collaboratively, particularly when B cells produce tumour-specific antibodies and cytotoxic T lymphocytes are activated, they orchestrate a potent anti-tumour immunity, thereby significantly improving the patient’s prognosis (Fig. 2) [19].

### Antigen presentation and differentiation of B cells

#### The multifaceted role of B cells as antigen-presenting cells in TLS

As antigen-presenting cells (APCs), B cells have the capacity to activate and proliferate into plasma cells (PCs) by specifically interacting with cognate antigens through B cell receptors (BCRs), initiating signaling cascades that lead to antigen internalization [55]. Within TLS, B cells can present internalized and processed antigens in association with major histocompatibility complex (MHC) II molecules to CD4 + T cells via the BCR, triggering their activation [52]. In ovarian cancer, B cells demonstrate the capability to present antigenic peptides to CD8 + T cells via MHC I molecules, providing evidence that antigen presentation by MHC I may be of significant importance [56]. Rita Cabrita et al. uncovered that individual B cells within TLS express high levels of both MHC class I and class II molecules, suggesting the proficiency of TLS B cells in antigen presentation [57]. Furthermore, Sheng Hong et al. utilized RNA phage Qβ-derived virus-like particles as a model antigen to show that antigen-specific B cells act as potent APCs, capable of activating naïve CD4 + T cells and encouraging their differentiation into T follicular helper (Tfh) cells [58]. Studies on NSCLC have revealed that tumour-infiltrating B cells present antigens and activate CD4 + T cells in certain NSCLC patients. The activation or depletion of TIL-B phenotypes correlates with CD4 + T cell phenotypes and is associated with improved clinical outcomes in NSCLC patients, showcasing elevated TME activation and enhanced TIL-B antigen presentation [59].

#### Differentiation of B cells into plasma cells for antibody production

Recent studies have demonstrated a significant correlation between the high density of TIL-B and PCs within TLS and improved clinical outcomes across various cancer types [60–62]. These PCs can generate tumour-specific antibodies that attach to tumour cells. These antibodies can block the activity of specific target proteins on tumour cells, trigger the complement system, and enhance both antibody-dependent cytotoxicity (ADCC) and antibody-dependent cellular phagocytosis (ADCP) [63]. Although the exact mechanism by which PCs and their antibodies in TLS contribute to cancer inhibition is not fully comprehended, a notable presence of B cells and PCs capable of producing IgG and IgA in TLS associated with ovarian cancer, melanoma, lung adenocarcinoma (LUAD), and NSCLC has been observed and found to be beneficial in improving the overall survival (OS) of patients [64–67]. This observation underscores their pivotal role in augmenting anti-tumour immunity. Mucosa-associated lymphoid tissue with active GCs was identified in 56% of lymphocyte-infiltrating tumours in surgical specimens of pancreatic ductal adenocarcinoma (PDAC). Additionally, restricted areas exhibited the B-cell lymphoma 6 (BCL6), CD21, and Ki67 expression, indicative of somatic hypermutagenesis and affinity maturation of GC B cells. The study found a rise in the proportion of IgG-expressing B cells in both intratumoral and peripheral blood of TLS patients. Analysing the BCR sequences of intratumoral TLS led to the identification of IgG1 subclass heavy chain as the predominant type, indicating that IgG1 class switching is favored in B cells within TLS during antigen recognition [68]. Additional investigations into PDAC have revealed that PDAC not only harbors mature TLS, enhancing T cell functionality and containing a diverse array of tumour-reactive T cells, but also facilitates the differentiation of B cells into PCs within these structures. This process is attributed to the orchestrated interactions among TGF-β signaling, CXCL13 expression by activated T cells, and the supportive role of TGF-β-producing fibroblasts, which collectively promote B cell activation and the sophisticated development of TLS [69].

In NSCLC, Claire and colleagues examined the level of differentiation of B cells located within the tumour using IHC. They found that all B cells present in the tumour had a stage of differentiation that was aligned with their in situ organization in TLS, as revealed by comparison with SLOs. IgG and IgA antibodiesagainst tumour antigens including LAGE-1, MAGEA1, MAGEC2, TP53, NY-ESO-1, and other MAGE antigens were discovered in the supernatants of NSCLC patient B-cells via enzyme-linked immunosorbent assay (ELISA) analysis, indicating the possibility of TLS as an active location for humoral immune responses [70]. In clear cell renal cell carcinoma (RCC), all stages of B-cell maturation leading to PC formation were identified by the researchers. The PC-related gene MZB1, along with the immunoglobulin genes IGHG1 and IGHA1, displayed high expression levels in both the TLS region and the tumour region away from the TLS [71]. This is a strong indication that in TLS, partially antibody-secreting PCs are generated after in situ antigen-driven B-cell activation, and have the ability to migrate to distant sites. Specific labelling of PCs and fibroblasts shows that the former align with fibroblast trajectories and embed within fibroblast networks, locally and further afield within the TLS region [71]. This data suggests that PCs can spread distally along fibroblast paths within the tumour bed (Fig. 2a).

In ovarian cancer, TLS is often surrounded by densely infiltrating PCs, who produce antibodies within the tumour. Their ability to target tumour-associated antigens (TAAs) has also been demonstrated. Although Iglesia and colleagues found that B-cell profiles were inversely associated with OS in glioblastoma and renal cancer via assessing several published B-cell/PC profiles for 11 cancer types, they also stated that bioinformatics methods still have limitations because different B-cell characteristics and other immune cell characteristics usually produce different prognostic outcomes in the same tumour type [72].

### T cells exert both cytotoxic and helper functions

During the anti-tumour immune response, APCs capture tumour antigens from tumour tissue and migrate to draining lymph nodes (DLNs), where they present these antigens to T-cells, triggering T-cell activation [73]. TA-TLS form in close proximity to malignant cells within the TME, facilitating a more direct and efficient T-cell activation compared to distant DLNs [74]. APCs can rapidly migrate into the TLS and present antigenic peptides to T cells, and the amount of tumour antigen in the TLS can exceed that in the tumour-draining lymph node (TDLN) [75]. Importantly, a study by Miao He et al. demonstrated that TLS maturation in NSCLC is linked to a lower risk of postoperative recurrence in patients, whereas tumour-invasive TDLN reduces the prognostic relevance of TLS [76].

Within the B-cell zone of TLS, GC B cells are closely correlated with total TILs, which are marked by Tfh TIL, T helper 1-directed CD4 TIL, TIL-B antibody secretion and long-term survival, indicating the existence of mature TLS [54, 77]. The principal accessory cell of TIL-B is closely linked to Tfh TIL, identified by expression of CXCR5, a marker known for its role in anti-tumour immune responses within TLS across various tumour types [78]. It’s crucial to note that Tfh cells, typically located within and adjacent to B-cell follicles, are indispensable for B-cell development and survival. They synergistically promote B-cell activation and differentiation by facilitating interactions between inducible co-stimulatory molecules such as ICOS:ICOS ligands (ICOSL) and CD40:CD40 ligand (CD40L), along with cytokines including IL-10 and IL-21. These interactions subsequently enhance the expression of Bcl6, thereby stabilizing the Tfh cell phenotype [10, 79, 80]. This mechanism aligns with observations from a CRC mouse model, where the introduction of Helicobacter hepaticus (Hhep) led to an increase in TLS numbers around the tumour, with the CD4 + T-cell response driven by Tfh cells playing a pivotal role. The absence of Tfh cells, however, resulted in the disappearance of TLS and diminished immune cell infiltration [81]. Thus, functional Tfh cells within TLS are key to effectively activating GC B-cells and facilitating the production of immunoglobulin antibodies, while also inducing the expression of IFN-γ and GZMB in activated CD8 + T-cells, enabling their activation and egress from the TLS [54].

Recently, researchers discovered two lymphocyte subsets that are age-dependent - CD153PD-1CD4 senescence-associated T (SAT) cells and CD30T-bet age-associated B-cells (ABCs) - in a mouse model of induced senescence for renal injury TLS. In this model, CD153 or CD30 gene deletion impairs the functional induction of SAT cells and reduces the number of ABC and GC B cells, thereby affecting TLS formation. This study also verified the presence of Tfh cells with similar SAT gene expression and CD153/CD30 signaling pathway in humans [82]. In addition, in mouse spleen age-associated CD4 + T cell subpopulations were also observed in the spleen of mice, including exhaustive cells (with gene expression similar to SAT cells), cytotoxic cells, and Treg cells [83]. This suggests that age-related changes in immune cell function may have broader systemic effects beyond renal injury, perhaps affecting cancer immunity.

## Follicular regulatory T (Tfr) cells and B cells interact in TLS to promote tumour progression

In the cancer immunity cycle, the balanced ratio of effector T cells to follicular regulatory T cells (Tfr) is critical, determining the outcome of cancer immunity. Tfr cells, characterized by the CD25 + CXCR5 + GARP + FOXP3 + phenotype and a demethylated forkhead box protein 3 (FOXP3) gene, undergo differentiation through multiple signaling pathways, primarily regulated by FOXP3 and Bcl-6 [50, 84]. The presence of Bcl-6 in Tfr cells, a key transcription factor also found in Tfh cells, suggests a functional link between Tfr and Tfh cells. Recent studies demonstrate that activation of IL-2, STAT3, and STAT5 pathways facilitates the conversion of memory Tfh cells into functional Tfr cells, targeting the FOXP3 and BCL6 genes [85]. Tfr cells produce high levels of TGF-β, which significantly inhibit Tfh cell expansion, GC activation of self-reactive B cells, and autoantibody production [86, 87]. However, mouse studies have demonstrated that IL-21 impedes Tfr cell development by inhibiting protein kinase B (Akt) phosphorylation and diminishing TGF-β and Foxp3 expression [85]. Consequently, the ratio of functional Tfh to Tfr cells dictates the varied impacts on tumour immunity [88]. In BC, Tfr cells have been observed to inhibit Tfh cell function within TLS [54]. Another study identified a significant presence of tumour-infiltrating regulatory T (Ti-Treg) cells around BC tumours, and these Ti-Treg cells were associated with recurrence and death in patients [89]. In NSCLC patients, Tregs migrating within TLS exhibited poor prognostic value, indicating that these TLS-associated Treg actively suppress the immune functions of certain cells such as DCs, T and/or B cells, mirroring findings in lung tumour models [90]. A negative correlation was observed in NSCLC between TLS-B cell density and DPP4 gene expression, a receptor implicated in TCR-mediated T-cell co-activation and Treg-mediated immunosuppression on TIL CD4 + T cells [67]. In early-stage lung cancer, an enrichment and infiltration of Tfr cells within intratumoral TLS were noted, with Tfr cells showing a negative correlation to CD8 + T cells [88].

The ultimate effect of B cells within a tumour is influenced in many ways, within the TLS, by their own phenotype, secreted cytokines, the type of antibody produced by differentiation into PCs, the presence and activation of Treg and activated macrophages [91, 92]. The presence of PD-1 in activated B cells inhibits BCR-mediated signalling, affecting self-maturation and antibody production. In turn, anti-PD-L1 antibodies acting on PD-L1 + Breg cells can impair their inhibitory effect on CD8 + and CD4 + T cells [93]. Although both IgG- and IgA-secreting PCs have been observed in RCC, only a small fraction of IgA is present, and effective anti-tumour activity is mainly associated with IgG [94]. IgA production may be associated with immunosuppressive TGF-β, as well as the secretion of inhibitory cytokines PD-L1 and IL-10 [52, 95]. This suggests that they are usually associated with a poor prognosis (Fig. 2b) [96]. Additionally, a high proportion of intratumoral IgA was observed to be associated with poor prognosis in LUAD [65]. Breg subsets, a subtype of B cells, can induce Treg cell differentiation and are associated with poor clinical outcomes in cancer [97]. Cytokines such as IL-10, IL-35, and TGF-β produced by Breg cells may indirectly inhibit the anti-tumour immune response on the one hand, and on the other hand, may also impair the effector function of T cells, tilting the macrophage towards an immunosuppressive phenotype [1]. This ultimately leads to an inefficient anti-tumour immune response. The presence of Breg cells in E-TLS-like lymphoid aggregates containing Treg cells was observed in BC, and their coexistence resulted in shorter metastasis-free survival compared to Treg cells alone [98].

Tumour-associated myeloid cells have been reported to be associated with poor prognosis. Tumour-associated macrophage-expressing PD-L1 can directly or indirectly suppress T cell responses by recruiting Tregs [99]. Additionally, macrophage-produced indoleamine 2,3-dioxygenase (IDO) has an inhibitory effect on immune cells [100]. M2-polarized macrophages are associated with poor cancer prognosis in BC, liposarcoma, and gastrointestinal stromal tumours [101–103]. The analysis of TLS in patients with colorectal liver metastases revealed that the density of Treg cells, M2 macrophages, and Tfh cells in intratumoral TLS was significantly higher than that in peritumoral TLS and positively correlated with poor survival [104]. In a rat model of lymphatic malformation (LM), M2 polarized macrophages may accumulate in infected LM via TLS and contribute to disease progression by secreting VEGF [105].

## TLS as biomarkers for tumour prognosis

Numerous studies have demonstrated a correlation between TLS and heightened objective response rates, indicating that TLS is a promising marker for immunotherapy. In light of TLS’s contribution to tumour immunity, researchers have increasingly explored the nature of TLS’s existence and its genetic signature to develop precise and dependable predictive biomarkers for cancer patients’ prognosis and treatment (Table 2).

**Table 2:** Prognostic effects of different TLS characteristics on different disease types

TLS features		Disease types	Methodology	Prognostic value	Experimental Subject	References
Quantity and density	High TLS density	iCCA	mIHC	Positive	Patients	[16]
		HCC	IHC	Positive	Patients	[106]
	Quantity	Non-functional Pancreatic neuroendocrine tumours	H&E IHC	No prognostic significance	Patients	[16]
Locations	Intra-tumoral	iCCA	mIHC	Positive	Patients	[16]
		CRC liver metastases	mIHC	Positive	Patients	[104]
		HCC	H&E IHC	Positive	patients	[49]
	Peritumoral	iCCA	mIHC	Negative	Patients	[16]
		Melanoma	mIHC	Positive	Patients	[107]
		HCC	H&E IHC	No prognostic significance	patients	[49]
Molecular or tissue characteristics	CXCL13	Bladder cancer	RNA seq	Positive	Patients	[108]
		Influenza A virus	Flow cytometry	Positive	Mice	[109]
	IL-22, IL-23	Crohn’s disease	H&E, RNA seq, Flow cytometry	Positive	Mice	[110]
	HEV	BC	IHC	Positive	patients	[49]
	ND	Bladder cancer	IHC	Positive	patients	[111]
	CD153/CD30	Chronic kidney disease	Flow cytometry, RNA seq	Negative	Mice	[82]
		Endothelial notch signaling	RNA seq, Flow cytometry, IF	Negative	Mice	[112]

mIHC: Multiplex immunohistochemistry; IHC: Immunohistochemistry; H&E: Hematoxylin and eosin staining; RNA seq: RNA sequencing; IF: Immunofluorescence

### The number, density and location of TLS affect tumour prognosis

The presence of TLS in solid tumours is generally associated with a favorable prognosis due to indicative effective immune infiltration. The significantly lower number of TLS in patients with stage III NSCLC compared to those with stage II suggests that tumour cells evade the immune response by dysregulating specific chemokine pathways to promote TLS formation during cancer progression [113, 114]. Moreover, lung cancer patients with chronic obstructive pulmonary disease (COPD) exhibited fewer B cells and TLS, correlating with earlier mortality [115]. Analysis in triple-negative breast cancer (TNBC) revealed that high-density PCs were associated with increased TLS counts and a better prognosis than in TNBC with low-density PCs [116].

Further investigations have shown that both peritumoral and intratumoral TLS are present in mice and humans, occasionally extending into normal tissues distant from the tumour [91]. It is currently unclear whether the difference in spatial location between intratumoral and peritumoral TLS holds predictive and/or prognostic value. The evidence available suggests that intratumoral TLS may have better prognostic significance, although this conclusion has yet to be confirmed by extensive analyses [117]. In BC, IHC analysis revealed that the presence of TLS adjacent to or distal from the tumour border correlated with a worse prognosis compared to those without peritumoral TLS, with an increase in the density of peritumoral TLS further exacerbating the prognosis [118]. Another study on BC revealed that TILs were observed at nearly all metastatic sites, whereas TLS were only identified in the liver and lungs, with their presence being almost negligible in brain metastatic lesions [119]. This study implies that the existence and structure of TA-TLS are connected to the TME and anatomical position, and that TLS tissue at diverse spatial levels is created by varied mechanisms that require further exploration in regards to their immunological properties and implications for patient survival and response to treatment.

### TLS detection and quantitative analysis

**Fig. 3:**
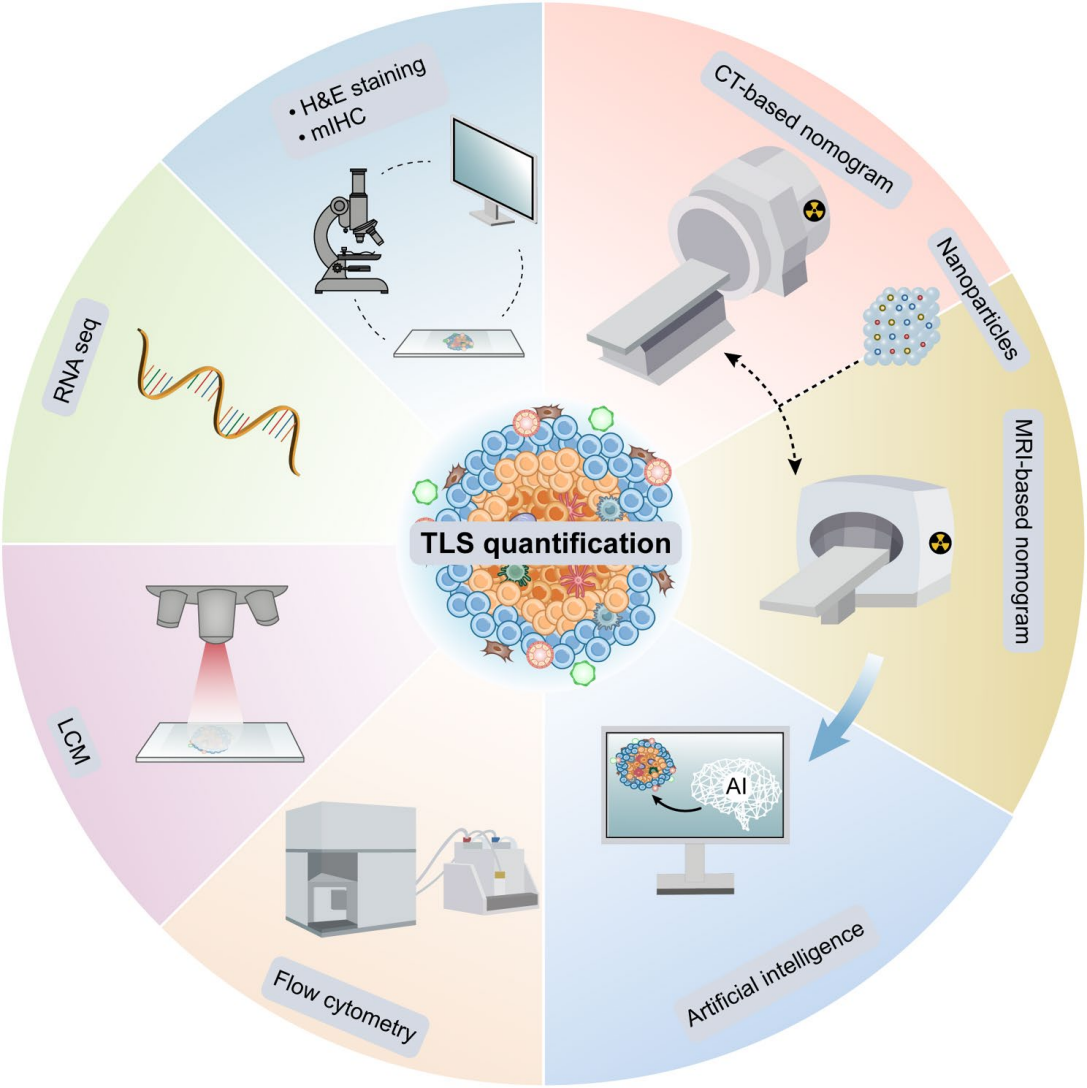
Strategies for quantitative analysis of TLS. TLS as a predictive marker for cancer detection and immune response can be detected and quantified by H&E staining, mIHC, Laser Capture Microdissection (LCM), spatial transcriptomics, and flow cytometry. The application of nanomaterials and artificial intelligence (AI) has led to advances in non-invasive imaging with nanoprobe-based CT and/or imaging. Imaging information can be further analyzed by AI algorithms to improve the efficiency and specificity of TLS detection

Current conventional methods for detecting TLS include hematoxylin and eosin (H&E) staining, IF, mIHC, and spatial transcriptomics [120]. These techniques rely on the examination of tumour samples from specific regions, hence the unavailability of surgically obtained samples due to inoperable conditions limits their effectiveness in TLS detection. The selection of specific circulating markers and/or the combined development and application of visual imaging characterization techniques may enhance the accuracy and specificity of TLS detection. Research in this area is ongoing (Fig. 3).

Quantitative assessment methods for TA-TLS can currently be divided into two main categories: genomic and histological analyses. However, histological analysis is prone to subjective interpretations, leading to potential bias. Given these disadvantages, histological analysis may not suffice for the efficient and accurate future assessment of TLS compared to genomic approaches [121]. A unique expression signature of nine genes (CD79B, CD1D, CCR6, LAT, SKAP1, CETP, EIF1AY, RBP5, PTGDS) was initially identified through differential gene expression analysis in melanoma, predicting prognosis and response to immunotherapy [57]. Similarly, in a cohort of 515 LUAD patients, these nine transcriptome-wide genes accurately predicted the presence of TLS, demonstrating that TLS characteristics serve as an independent positive prognostic factor for LUAD patients [122]. To quantify TLS, researchers calculated a TLS feature score based on the average expression levels of these nine genes. After determining the optimal threshold for high and low TLS feature scores using X-tile software, they correlated these scores with overall survival rates in patients. Patients with higher TLS feature score expression levels were categorized into a high TLS group, indicating a stronger presence of TLS within the tumour [123]. Not coincidentally, differential gene expression analysis of endometrial carcinoma (EC) showed that TLS was associated with CD8 + T-cell infiltration and L1CAM overexpression, which was only present in morphologically mature TLS with GCs [124]. As previously mentioned, stromal cells are a key factor in the formation of TLS. IFN induces the expression of chemokines including CXCL13 and CXCR5 in lung fibroblasts, thereby promoting the formation of TLS [109]. The expression of CXCL13, associated with tumoural TLS, serves as a potential biomarker for TLS presence in bladder cancer and may predict patient responsiveness to ICI in advanced stages of the disease [108].

Imaging histology, as a non-invasive imaging method, has shown great potential in the diagnosis and treatment of tumours by combining learning models with digital pathology [125]. It has been reported that in patients with NSCLC and intrahepatic cholangiocarcinoma (ICC), it is possible to combine the imaging features of computed tomography (CT) or magnetic resonance imaging (MRI) with clinical information for tumour diagnosis, efficacy evaluation, and prediction of molecular markers [126, 127]. Emerging research suggests the potential of combining these imaging features with advanced learning models and digital pathology for enhanced TLS visualization. A recent study on LUAD revealed that lung nodules with a more solid portion were associated with a high TLS density by CT scanning [128]. Preoperative CT imaging histograms were utilized to predict TLS status and recurrence-free survival in ICC patients, proving more effective than standalone imaging histograms or clinical models in assessing intratumoral TLS status [129]. Researchers developed a graphical whole-slide imaging (WSI) method for assessing lymphocyte aggregation, enabling differentiation between TLS and other infiltrates by analyzing CT imaging characteristics [130]. It is crucial to note that CT imaging primarily reveals higher-density lymphocyte aggregates, rather than the distinctive structures of TLS. While imaging results align with histopathological findings of TLS locations, they offer correlative predictions rather than definitive insights. Furthermore, exploratory technologies, including the deployment of nanoprobes for identifying TLS-specific markers in imaging scans, signal significant advancements in the realm of immunological imaging. Li et al. identified CT imaging characteristics indicative of TLS, enabling preoperative, non-invasive TLS prediction in HCC [131]. In a study employing needle optical coherence tomography (OCT) for the assessment of LNs, researchers considered the region of rapid signal decay to be the GC and minimized the imaging probe to look at B cells and the GC in the internal nodes, which theoretically allows imaging of the TLS [132]. Although promising, these innovative techniques remain in the experimental phase and necessitate further validation before being deemed fully developed.

Advancement in Artificial Intelligence (AI) technology has introduced innovative means to conduct deep learning on graphical data. These techniques can be combined with image features to facilitate a novel method of recognizing TLS. HookNet, a segmentation model for histopathological WSIs, can accurately identify GC-containing TLS by leveraging recognition of previously identified TLS [133]. This methodology has already been implemented in the detection of lung cancer. Additionally, TESLA, a machine learning framework for annotating tissue at the pixel level in spatial transcriptomics, enables the direct annotation of immune and tumour cells in histological images and can further detect TLS in TME [134]. The future integration of AI with genomics, spatial genomics, and immuno-scoring could enable the development of robust TLS quantification methods. These methods have the potential to be applied across a wide range of cancer types, ensuring stability in gene-level quantification.

## Preclinical induction of TLS and prospects for its application

The presence of TLS and B cells across a range of tumour types, including carcinomas, melanomas, soft tissue sarcomas, and RCC, is predictive of a positive response to ICB therapy, indicating the potential for ICB to assume an enhanced role in the treatment of tumours rich in B cells and TLS [11, 17, 57, 135, 136]. Furthermore, plasmacytoid B-cell profiles and TLS-associated B-cell profiles have been found to have a positive correlation with ICB responses in melanoma and LUAD [107, 137–139]. To improve clinical outcomes post-ICB, therapies supporting TLS induction and activating B cells are necessary and require further research.

**Fig. 4:**
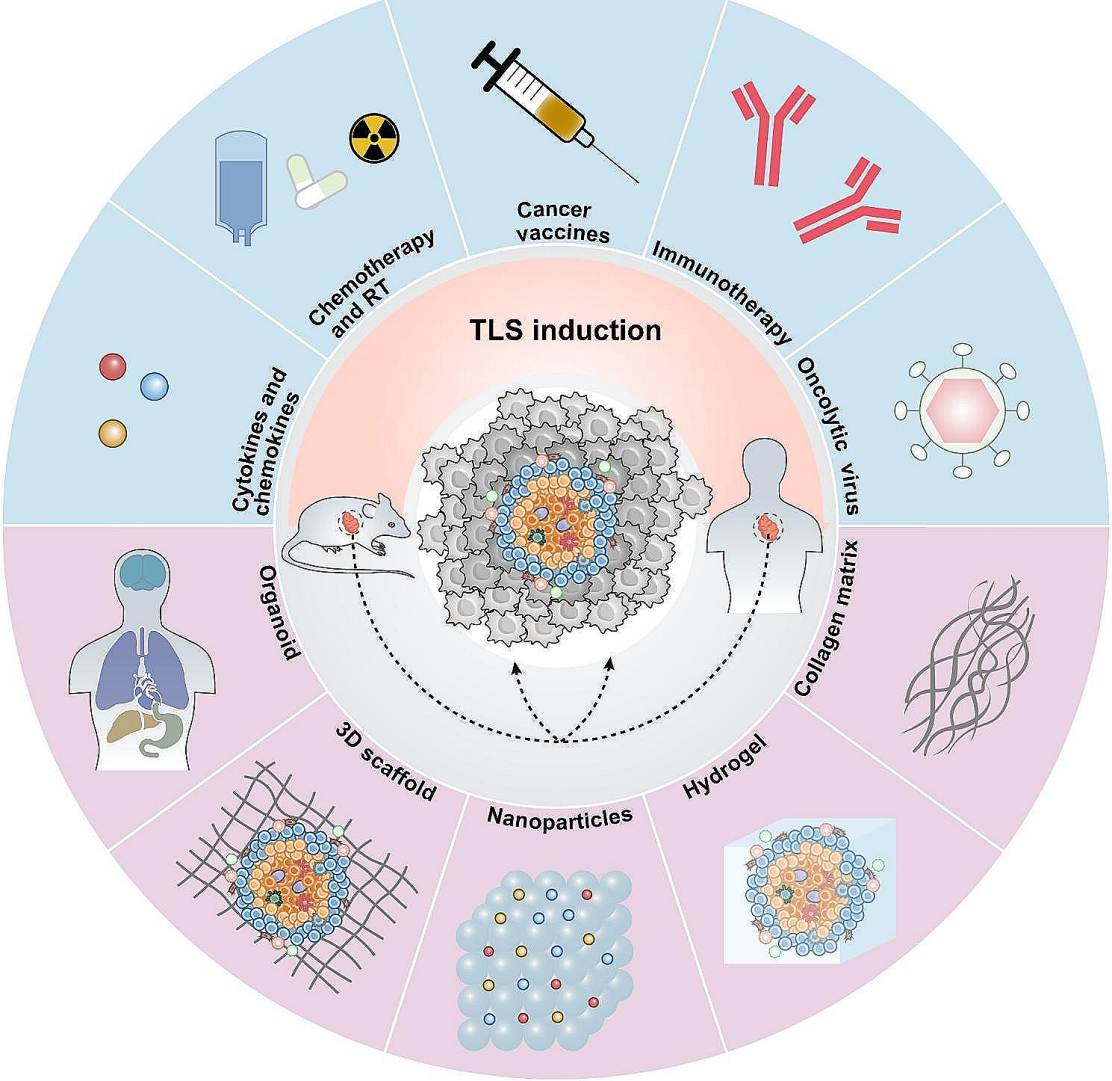
Methods and prospects for inducing TLS in mice and cancer patients in vivo The emergence of TLS has been induced in the past in mice or cancer patients by means of cytokines and chemokines, chemotherapy, radiotherapy, cancer vaccines and immunotherapy. Recently lysosomal viruses (OV) have also raised possibilities in TLS induction. In addition to this, biomaterials show considerable promise for TLS induction. Collagen matrices, hydrogels, nanomaterials, three-dimensional (3D) scaffolds, and organoids all have the potential to serve as new induction modalities

In mouse models of cancer and in patients with cancer, cytokines and chemokines, chemotherapy, radiotherapy, cancer vaccines and ICB treatments have demonstrated the ability to induce intra-tumour formation of TLS and increase the infiltration of immune cells (Table 3). In pancreatic cancer, targeted LIGHT localizes to the tumour vasculature via vascular-targeting peptide (VTP), inducing vascular normalization and the formation of HEV and TLS through the self-amplifying cycle of pancreatic cancer. The mechanism may be that LIGHT stimulates macrophages to express the inflammatory cytokines CXCL13, CXCL21, TNF, and IL-6, which recruits T-cells and thus promotes TLS formation [140, 141]. Low doses of STING agonists enhance T-cell and DC infiltration, foster an inflammatory TME, and stimulate DC maturation, which leads to the local secretion of CCL19, CCL21, LTα, LTβ, and LIGHT, thereby promoting vascular normalization and TLS formation [142]. In a trial of 39 patients with resected PDAC treated with a GM-CSF vaccine, 33 exhibited TLS aggregates within two weeks of vaccination. And inhibition of the Treg signalling pathway and enhancement of Th17 signalling within these TLS aggregates were found to be associated with increased survival [143]. Analysis of TLS in pancreatic cancer patients undergoing preoperative neoadjuvant chemoradiotherapy (NAC) through IHC revealed altered TME compositions, increased immune cell proportions, and favorable prognoses [144]. However, a study on lung squamous cell carcinoma (LSCC) patients indicated that corticosteroid use during chemotherapy impacted GC and TLS formation [47]. It is crucial to acknowledge that definitive evidence supporting the capacity of certain immunotherapies to initiate TLS in cancer is presently insufficient, necessitating further empirical research. Specifically, although radiotherapy has been observed to trigger the formation of intratumoral TLS, this phenomenon primarily results from the immediate depletion of local immune cells caused by low-dose radiation, accompanied by a diminution in the size of pre-existing TLS, which typically recuperates within a fortnight [145, 146]. Hence, induction of TLS by radiotherapy is currently not supported by sufficient evidence. Importantly, conventional cancer immunotherapies exhibit limitations, such as varying systemic toxicities and heterogeneous therapeutic outcomes, depending on the type of immunotherapy employed [7, 147]. Recent advancements in nanotechnology and biomaterials have unveiled significant potential for innovation in cancer immunotherapy (Fig. 4) [148].

**Table 3:** Methods for inducing TLS

Therapy	Treatment	Cancer type	Experimental Subject	Identification Method	TLS Change	References
Using cytokines or chemokines	CXCL13, CCL21	PDAC	Mice	IF, IHC	increase	[149]
	LIGHT-VTP	Insulinoma	Mice	H&E, IHC, IF, flow cytometry	increase	[140]
	STING agonists	melanoma	Mice	IF, flow cytometry	increase	[150]
	CD40 agonists	glioma	Mice	H&E, IHC, IF, flow cytometry, RNA-seq	increase	[151]
Cancer therapies	Radiation Therapy	LUAD	Mice	H&E, IHC	increase	[145]
	neoadjuvant chemotherapy	Hepatoblastoma	12 patients	H&E, IHC, RNA-seq	increase	[152]
		epithelioid mesothelioma	138 patients	H&E	increase	[153]
		lung cancer	122 patients	H&E, IHC	UC (unchanged)	[154]
		urothelial cancer	24 patients	H&E, IHC, IF, RNA-seq	increase	[155]
	Maintenance immunotherapy	Melanoma, RCC	34 patients	H&E, IHC, IF, RNA-seq, flow cytometry	UC	[17]
	HPV 16 vaccine	cervical	39 patients	H&E, IHC, RNA-seq	increase	[156]
	pancreatic tumour vaccine	pancreatic cancer	12 patients	H&E, IHC, RNA-seq, flow cytometry	increase	[143]

VTP: vascular targeting peptide; IF: Immunofluorescence; H&E: Hematoxylin and eosin staining; IHC: Immunohistochemistry; RNA seq: RNA sequencing

### Collagen matrix

The formation of collagen in mammalian connective tissue requires intricate intra- and extracellular processes. Its bioactivity arises from its complex structure, which has facilitated its recent biosynthetic applications [157]. In mice, the creation of TLS was initially achieved by implanting artificial collagen matrices derived from a thymic stromal cell line expressing LTα, producing functional TLS [158]. Subsequent implantation of these structures into severely combined immunodeficient mice resulted in a robust secondary immune response and production of high levels of high-affinity IgG antibodies [159]. Similarly, collagen sponge scaffolds containing a combination of chemokines CCL19, CCL21, CXCL12, CXCL13 and soluble RANKL as well as LTα1β2 were implanted into mouse kidneys, and TLS were successfully constructed containing isolated B and T cell regions as well as HEVs [160].

### Hydrogel

Hydrogels are unique entities consisting of a crosslinked polymer mesh in three dimensions, capable of extending the duration of drug effectiveness owing to their favourable biological characteristics like biocompatibility, water retention, and bioadhesive qualities, rendering them an exceptional therapeutic vehicle [161]. According to a study by Alberto Purwada et al., hydrogels with stromal cells expressing BAFF and IL-4 enhanced GC responses, facilitating antibody class switching events [162]. Recently, researchers have constructed an interferon gene activator hydrogel that initiates and activates the stimulator of interferon genes (STING) and TLR-9 pathway to enhance TLS formation [163].

### Nanomaterials

As transport carriers, nanomaterials can transport biologically active combinatorial loads to specific sites in the body in a controlled manner and through their high specificity, thereby effectively reducing dose and off-target toxicity [164]. The combination of nanomedicine and immunotherapy has been effective in enhancing anti-tumour immune responses and improving the safety of therapeutic responses [165]. In a study simulating a mouse model of nasopharyngeal carcinoma, researchers developed a nanovaccine composed of Epstein-Barr virus (EBV) nuclear antigen 1 (EBNA1) and tannic acid, combined with Mn2 + and cytosine-phosphate-guanine (CpG) as dual adjuvants. This formulation synergistically activates DCs, B cells, and T cells through the TLR-9 and STING signaling pathways. Additionally, it enhances the secretion of CCL19/CCL21, CXCL10, and CXCL13 in the TME through the activation of the LT-α and LT-β pathways, which together promote the recruitment of peripheral immune cells, thereby promoting the formation of TLS, enhancing local immune response and delaying tumour growth [166]. The ‘Nano-sapper,’ a biomaterial comprising an anti-fibrotic core of phosphate-modified α-mangostin and a plasmid encoding the immune-boosting cytokine LIGHT, has been demonstrated to promote TLS formation and impede tumour growth. This biomaterial achieves its effects by reversing the abnormal activation of fibroblasts, diminishing collagen deposition, normalizing the vasculature within tumours, and enhancing the local expression of chemokines by lymphocytes. These actions lead to increased infiltration of CTLs, the induction of TLS within tumours, the remodeling of the TME, and an improved efficacy of ICB therapy [167].

### 3D technology and organoid

Three-dimensional (3D) scaffolds, fabricated via 3D printing technology, offer a superior delivery mechanism for cancer vaccines over hydrogel scaffolds, attributed to their outstanding porous structure and biosafety. Studies have demonstrated that these 3D scaffolds, when loaded with immunomodulators, can recruit and activate numerous immune cells, particularly APCs and T cells, mimicking a lymphoid organ’s function. This process facilitates the development of an “artificial tertiary lymphoid structure,” proven to inhibit tumour growth effectively [168].

Human organoids have enormous potential for biomedical applications in organ development and disease onset, preclinical drug development, and regenerative medicine [169]. Organoids are essentially multicellular clusters formed in vitro by 3D cell culture techniques, giving us a possible model in terms of TLS induction [170]. Küçükköse E and colleagues developed a humanized mouse model for spontaneous multi-organ tumour metastasis using patient-derived organoids. The study found that ICB therapy successfully eradicated liver metastases without affecting peritoneal metastases, which, contrary to the shrinking primary tumour, increased after therapy cessation, with B-cell infiltration and TLS formation observed at the primary tumour and liver metastasis sites but not at peritoneal metastases, potentially explaining the variability in ICB treatment outcomes [171].

Nonetheless, the clinical application of organoid model construction is limited due to its low success rate, difficulty in blood vessel formation, large repeatability and variation, and difficulty in cost control [172]. Recent studies combining organoids with 3D bioprinting and organ vaccines have opened up new possibilities for precision research in cancer modelling and have addressed some of the dilemmas and limitations encountered in organoid research. 3D bioprinting and organ microarrays can generate or mimic the vasculature system to supply nutrients to organoids, allowing for greater growth and development [172, 173]. Goyal et al. also found that T and B lymphocytes isolated non-invasively from human peripheral blood can spontaneously assemble into ectopic lymphoid follicles in a dual-channel organ-on-chip microfluidic control system [174]. These have inspired new ideas for TLS induction strategies.

Through the elaboration of conventional cancer immunotherapy as well as methods developed on the basis of nanotechnology and biomaterials to induce TLS, it can be concluded that this mechanism of TLS induction may be formed through a network mechanism in which multiple cells, multiple chemokines and multiple signalling pathways act together. Although the intermediate effects produced by the different induction strategies are not identical, they all seem to attenuate the immunosuppressive effects in TME while boosting the function of immune cells, thus activating the signalling cascade pathways for TLS production.

## Conclusion and perspectives

The spatial structure of TLS (presence of GCs), its cellular composition (high-density TIL infiltration and differences in immune cell phenotypes), and its relationship to tumour location (peri-tumour or intra-tumour) affect the prognosis of tumour patients [3, 4, 25, 48, 175]. Notably, the dual role of TLS in tumour immunity—termed the “double-edged sword” effect—may hinge more on the proportions of various immune cell subpopulations within the TLS, particularly given the observed variability in T and/or B cell phenotypes. Among T cell subsets, CD4 + T follicular helper (Tfh) cells, expressing high levels of CXCR5 and Bcl6, along with CD8 + T cells, are the primary contributors to anti-tumour immunity among T cell subsets. But the precise mechanism underlying the cytotoxic effect of TLS-modified CD8 + T cells on tumour cells remains unclear. Tfh cells facilitate B cell recruitment through the high expression of co-stimulatory receptors (ICOS and CD153), thereby slowing tumour progression. Tfr cells, characterised as CD25CXCR5GARPFOXP3, inhibit Tfh cell activity with the involvement of TGF-β. Despite the lack of consensus on markers for Breg cells, their secretion of immunosuppressive cytokines such as IL-10, IL-35, and TGF-β can inhibit effector T cell activity and promote the expansion of Treg cells, thereby facilitating tumour growth. The characterization of B cell subpopulations through RNA sequencing (RNA-seq) or specific antibodies revealed that, with the exception of Breg cells, all B cell subpopulations generally correlate with positive clinical outcomes. The in situ activation of GC-B cells within TLS for differentiation and antibody production further underscores the significance of B cells within the cancer immune cycle. The phenotype of different immune cell subpopulations is largely dependent on cytokines with different immune properties in the TME, as well as on the influence of cell signalling pathways, which are not yet fully understood.

As a novel specific biomarker to influence and determine the prognosis of cancer patients, the future development of TLS involves two main aspects. Although biomarkers of TLS have been widely studied, there is no established uniform standard for quantification of TLS in different patients and different cancers. First, we need to focus on the difficulties that the heterogeneity of TLS poses for the quantification and assessment of different cancer types. The combined application of specific TLS markers, imaging technologies and AI in histopathology may open up a new path for TLS detection and quantitative analysis.

Second, we need to effectively induce the occurrence and maturation of TLS in tumours. Traditional induction methods through injection of cytokines or chemokines, radiotherapy, chemotherapy, ICB therapy and cancer vaccines have successfully induced TLS in mouse models or humans, but novel biomaterials such as collagen mechanisms, hydrogels, nanomaterials as well as the development of organoid and 3D technologies have reinvigorated TLS induction and enhanced the induction efficiency. The possibility of using complex technologies, including novel biomaterials, to generate inducible TLS, is being investigated with very promising results in preclinical models [176]. Nevertheless, the translation of these technological tools into the clinic still needs to address issues of tissue safety, cost control and selection of appropriate humanised in vivo models. This is essential to guide further precision and efficiency of TLS in immunotherapy and prognosis.

## Data Availability

No datasets were generated or analysed during the current study.

## Abbreviations

3D: three-dimensional
ABCs: Age-associated B-cells
ADCC: Antibody-dependent cytotoxicity
ADCP: Antibody-dependent cellular phagocytosis
AI: Artificial Intelligence
APCs: Antigen-presenting cells
BC: Breast cancer
Bcl6: B-cell lymphoma 6
BCRs: B cell receptors
Breg: Regulatory B cells
C1q: Complement component 1q
CAF: Cancer-associated fibroblast
CCL: C-C motif chemokine
COPD: Chronic obstructive pulmonary disease
CpG: Cytosine-phosphate-guanine
CRC: Colorectal cancer
CT: Computed tomography
CTLA-4: Cytotoxic T - Lymphocyte Antigen 4
CXCL: C-C motif chemokine
DCs: Dendritic cells
DLNs: Draining lymph nodes
EBNA1: EBV nuclear antigen 1
EBV: Epstein-Barr virus
EC: Endometrial carcinoma
ELISA: Enzyme-linked immunosorbent assay
E-TLS: Early TLS
FAP: Fibroblast activation protein
FDCs: Follicular dendritic cells
F-IHC: Fluorescence immunohistochemistry
FOXP3: Forkhead box protein 3
GC: Germinal centers
H&E: Hematoxylin and eosin staining
HCC: Hepatocellular carcinoma
HEVs: High endothelial venules
Hhep: Helicobacter hepaticus
HNSCC: head and neck squamous cell carcinoma
HPV: Human papillomavirus
ICAM1: Intercellular adhesion molecule 1
ICB: Immune checkpoint blockade
ICC: Intrahepatic cholangiocarcinoma
iCCA: Intrahepatic cholangiocarcinoma
ICOS: Inducible co-stimulatory molecules
ICOSL: Inducible co-stimulatory molecular ligands
IDO: Indoleamine 2,3-dioxygenase
IF: Immunofluorescence
IFN: Interferon
IHC: Immunohistochemistry
IL: Interleukin
ILCs: Innate lymphocytes
ILFs: Isolated lymphoid follicle
IrAE: Immune-related adverse events
LCM: Laser Capture Microdissection
LM: Lymphatic malformation
LNs: Lymph nodes
LSCC: Lung squamous cell carcinoma
LTi: Lymphoid tissue inducing cells
LTo: Lymphoid tissue-forming cells
LTα1β2: Lymphotoxinα1β2
LTβR: LTβ receptor
LUAD: Lung adenocarcinoma
LVs: Lymph vessel
MADCAM 1: Mucosal vascular addressin cell adhesion molecule 1
MDSC: Myeloid-derived suppressor cells
MHC: Major histocompatibility complex
MIHC: Multiplex immunohistochemistry
MRI: Magnetic resonance imaging
NALT: Nasal associated lymphoid tissue
NKT: Natural killer T cells
NSCLC: Non-small-cell lung cancer
OCT: Optical coherence tomography
OS: Overall survival
OV: Lysosomal viruses
PCs: Plasma cells
PD-1: Programmed cell death protein 1
PDAC: Pancreatic ductal adenocarcinoma
PD-L1: Programmed cell death ligand 1
PFL-TLS: Primary follicular-like TLS
RANK: NF-κB receptor activator
RANKL: NF-κB ligand receptor activator
RCC: Renal cell carcinoma
RNA-seq: RNA sequencing
SAT: Senescence-associated T-cells
SCID: Severely combined immunodeficient
SFL-TLS: Secondary follicle-like TLS
SLOs: Secondary lymphoid organs
STING: Stimulator of interferon genes
TAAs: Tumour-associated antigens
TA-TLS: Tumour-associated tertiary lymphoid structure
TCRs: T cell receptors
TDLN: Tumour-draining lymph node
Tfh: T follicular helper cells
Tfr: Follicular regulatory T cells
TGF: Transforming growth factor
Th: T helper cells
TIL-B: Tumour-infiltrating B-cell
TILs: Tumour-infiltrating lymphocytes
Ti-Treg: Tumour-infiltrating regulatory T
TLR9: Toll-like receptor 9
MyD88: Myeloid differentiation factor 88
TLSs: Tertiary lymphoid structures
TME: Tumour microenvironment
TNBC: Triple-negative breast cancer
TNF: Tumor necrosis factor
Treg: Regulatory T cell
VAMC: Vascular smooth muscle cells
VCAM 1: Vascular adhesion molecule 1
VEGF: Vascular endothelial growth factor
VTP: Vascular targeting peptide
WSI: Whole-slide image
